# Polyion complex (PIC) particles: Preparation and biomedical applications

**DOI:** 10.1016/j.eurpolymj.2016.06.003

**Published:** 2016-08

**Authors:** Ignacio Insua, Andrew Wilkinson, Francisco Fernandez-Trillo

**Affiliations:** School of Chemistry, University of Birmingham, B15 2TT, UK

## Abstract

Oppositely charged polyions can self-assemble in solution to form colloidal polyion complex (PIC) particles. Such nanomaterials can be loaded with charged therapeutics such as DNA, drugs or probes for application as novel nanomedicines and chemical sensors to detect disease markers. A comprehensive discussion of the factors affecting PIC particle self-assembly and their response to physical and chemical stimuli in solution is described herein. Finally, a collection of key examples of polyionic nanoparticles for biomedical applications is discussed to illustrate their behaviour and demonstrate the potential of PIC nanoparticles in medicine.

## Graphical abstract

**Figure:**
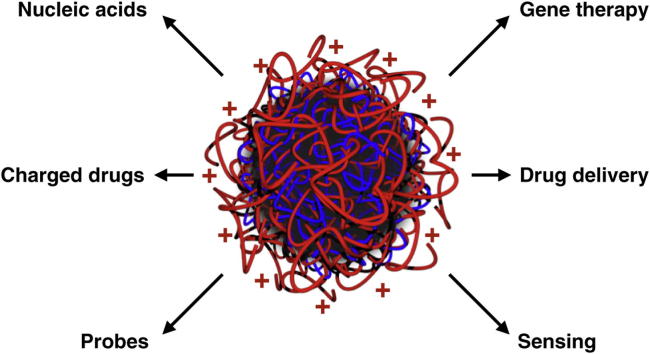

## 1 Introduction

Macromolecules carrying multiple charges (*a.k.a.* polyelectrolytes) are ubiquitous in nature and include nucleic acids, proteins or polysaccharides. The presence of these charges often dictates the physical properties of these biopolymers, including their self-assembly or their solubility in physiological environments. More importantly, charge-charge interactions often mediate relevant biological processes, from the package of DNA around histones [1], to the initial adhesion of microbes to surfaces and hosts [2]. It is not surprising then that researchers have tried to imitate these important polyionic interactions to develop synthetic systems that can interact with biology. As such, polyionic complexes (PIC), *a.k.a.* polyelectrolyte complexes (PECs) [3] or interpolyelectrolyte complexes (IPECs) [4], have been investigated as novel platforms to stabilise and deliver drugs [5], [6], proteins [7], [8] or nucleic acids [9], [10], [11].

The morphology of the complexes formed depends on the topology of the polyelectrolytes employed and the way these are assembled. For instance, ionic-hydrophilic block copolymers have been used for the preparation of PIC micelles [12], [13] and PIC vesicles (PICsomes) [14], while the sequential assembly of polyelectrolytes on surfaces and colloidal templates has been exploited for the preparation of layer-by-layer films [15] and capsules [16] respectively.

In this review we will focus on the preparation and biomedical applications of PIC particles, formed by the neutralisation of non-stoichiometric mixtures of polyelectrolytes. Other morphologies and their biomedical applications have been extensively reviewed elsewhere and interested readers are encouraged to read that literature [12], [13], [14], [15], [16], [17].

## 2 Self-assembly of PIC nanoparticles

**Fig. 1:**
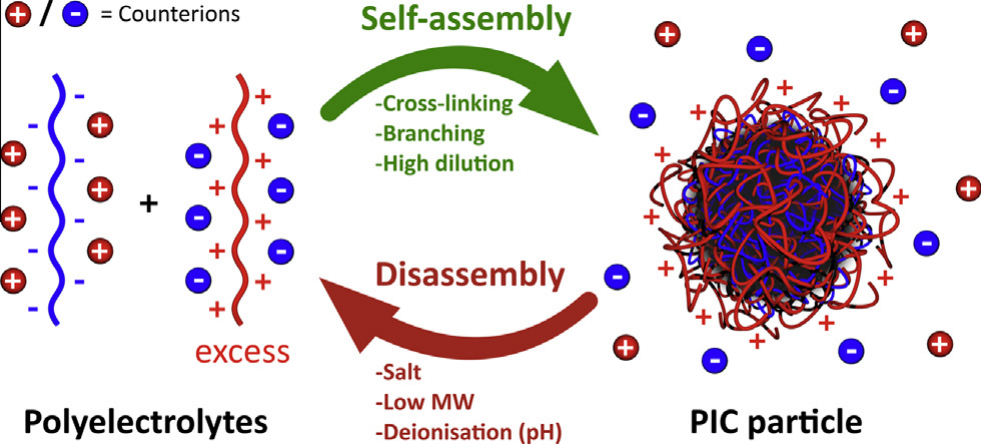
Representation of polyion self-assembly into PIC nanoparticles and subsequent counterion release (charged spheres). In this example, the excess of polycation in the mixture leads to the formation of a neutral core surrounded by the excess of cationic material.

The preparation of PIC particles was first reported by Fuoss and Sadek in the late 1940’s as a straightforward turbidimetric study of polyion condensation [18]. Over the last decades, more sophisticated instrumentation has allowed a deeper understanding of PIC particle assembly. It is now well established that non-stoichiometric mixtures of oppositely charged polyions lead to the formation of PIC particles, which consist of a neutral core entrapping 1:1 mixtures of oppositely charged polymers surrounded by a shell of excess polyion chains (Fig. 1) [19], [20]. The charged corona stabilises the coacervate by repelling other PIC particles in solution. In contrast, stoichiometric mixtures of polyions lead to unstable shell-deficient PIC particles, which flocculate due to the hydrophobic attraction between neutral coacervates [21], [22].

General models rationalise that PIC formation is primarily based on the electrostatic attraction between oppositely charged polyions [3], [18], [23], [24]. However, it has been demonstrated that the increase in entropy upon counterion release from polyelectrolytes is the main driving force in PIC particle self-assembly [25], [26].

PIC particles are often polydisperse systems with diameters comprised between 10 nm and 1 μm. Particle size is usually measured from indirect techniques, mainly static and dynamic light scattering, which maintain the integrity of the nanoparticles in solution. Imaging techniques are often used to support the results from light scattering, but it must be noted that in this case the size of PIC particles can be affected during sample preparation and analysis (especially in the case of drying and staining) [27].

The self-assembly of PIC particles is a reversible process where nanoparticles coexist in equilibrium with free polyions in solution (Fig. 1) [26], [28]. This dynamic nature of PIC particles inherently compromises their integrity upon dilution and addition of other charged species, which can cause electrostatic shielding and polyion exchange from these nanomaterials [29]. As a result, the stability of PIC particles can be seriously compromised in biological settings, where high concentrations of small electrolytes and charged proteins can be found [30]. However, PIC particles can be easily stabilised by cross-linking of the polyelectrolyte components, minimising this way the exchange with free polymers in solution (see Section 2.7). On the other hand, the structural damage caused by physiological electrolytes to PIC particles can be beneficial, for example by increasing the permeability of these nanomaterials thus triggering the release of preloaded cargos [31], [32].

Finally, it must be highlighted that only certain polyelectrolytes, mixed under suitable conditions, lead to disperse and stable PIC particles. Several factors need to be optimised during the formulation of these nanomaterials, in particular molecular weight, concentration and charge density of the polyelectrolytes, pH and ionic strength of the (aqueous) media, or relative ratio and mixing order of both polyelectrolytes. These parameters are normally interdependent, and have to be optimised in parallel. Often, stable colloidal PIC particles are therefore prepared at high dilution and low ionic strength, with an excess of one of the polyions, ideally with weak ionic groups in one of the polymers and a considerable mismatch in their molecular weights [28], [33]. Under different conditions, coacervates separate from solution and coalesce into dense materials known as *compact polyelectrolyte complexes*, which have also shown great potential in some biomedical applications such as synthetic cartilage and enzymatically active biocomposites [34]. To give the reader a deeper understanding of the variables affecting the complexation of polyelectrolytes, we will now describe how these factors affect PIC particle formation, explaining their fundamentals and influence on the characteristics of PIC particles.

### 2.1 Charge density, molecular weight and topology

The binding properties of polyions to other charged species show a strong dependency on the density and number of charged groups in the polyelectrolyte’s structure. As a result of the cooperative nature of polyelectrolyte binding, polymers with higher charge densities show stronger avidity for charged substrates.

The simplest way to increase charge density is to increase the polyelectrolyte’s molecular weight. Given the partially entangled conformation of polymers in solution [35], higher molecular weight leads to larger polyelectrolyte cores. This molecular weight dependency was nicely demonstrated by Fischer et al. with a comparative study on the preparation of DNA polyplexes from poly(diallyldimethylammonium chloride) (PDADMA) of four different molecular weights (5–244 KDa) [36]. Higher charge densities, corresponding to higher PDADMA molecular weights, formed more stable polyplexes as indicated by the higher tolerance of complexed DNA to ultrasonication and enzymatic digestion. The authors explained these results from the possible rearrangement of polyions with low charge densities, weakly bound to the polyplex. It has been rationalised that the stability of PIC particles is influenced by the number of charged groups per polymer chain (*i.e.* multivalency), and above an ‘upper critical length’ polyions can form stable complexes [37]. Nonetheless, a significant difference in the molecular weight of the polyions is often required for the colloidal stability of PIC particles [38].

Alternatively, charge density can be “diluted” by the addition of neutral monomers to the polyelectrolyte. Dautzenberg et al. evaluated the formation and stability of PIC particles from poly(styrene sulfonate) (PSS) and four PDADMA copolymers of comparable molecular weight, which contained increasing amounts (0, 25, 47 and 76%) of neutral *N*-methyl-*N*-vinylacetamide (p(DADMA)-co-(NMVA)) [21]. As the NMVA content in the copolymers increased (lower charge density), bigger particles formed with up to twice the size of those from 100% PDADMA. In addition, the destabilising effect of NaCl on PIC particles was significantly reduced with higher PDADMA contents. Nanoparticles prepared from pure PDADMA remained unaffected by concentrations of NaCl twice higher than those causing particles with weaker charge densities to swell. These results reinforce the direct correlation between charge density and the binding avidity of polyelectrolytes.

The topology of polyelectrolytes can also have a profound effect on their charge density. It is known that branched and dendritic polyelectrolytes show increased binding affinities to charged surfaces compared to their linear analogues [24]. This effect is due to the higher spatial density of monomers in branched structures and thus results in an increase in avidity. As such, branched polyelectrolytes can electrostatically cross-link multiple counter-polyion chains, creating tighter polymeric networks [39]. Dunlap et al. showed that, under the same conditions, whereas linear poly(ethylene imine) (PEI) formed polyplexes of 20–50 nm and left uncomplexed DNA fragments, branched PEI of similar molecular weight fully condensed DNA and formed significantly larger complexes (100–200 nm), probably due to interparticular condensation as a result of its higher binding capacity than linear PEI [40]. The high charge density of branched PEI is responsible for its remarkable capacity to tightly complex DNA, thus setting a benchmark for other transfection agents [41].

**Fig. 2:**
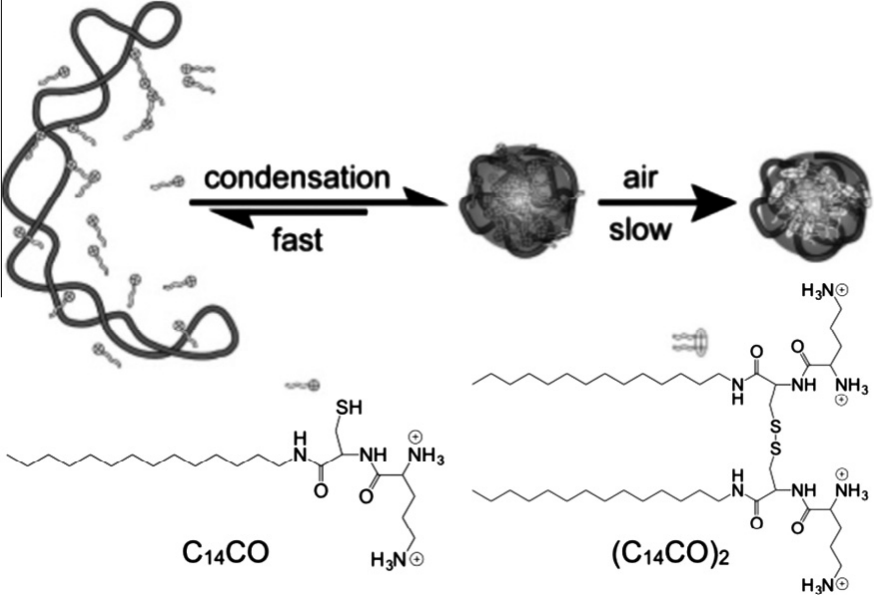
Plasmid DNA is condensed into monomolecular particles with a thiol-containing cationic detergent. The particles are stabilised by spontaneous oxidation of the detergent. Adapted from Ref. [43].

Although challenging due to their low charge density, PIC particles have been successfully prepared from small molecules bearing certain motifs that aid the nucleation process. For example, the Behr group studied the self-assembly of small cationic detergents with DNA into lipidic polyplexes or lipoplexes [42]. The authors prepared a dimerisable detergent (C_14_CO) that could condense DNA despite its low charge density, aided by the hydrophobic interactions between aliphatic chains (Fig. 2). After the initial aggregation of C_14_CO with plasmid DNA, the oxidative dimerisation of this detergent into (C_14_CO)_2_ caused a drop in its critical micellar concentration, thus increasing the packing and stability of the complex.

### 2.2 Polyelectrolyte concentration

**Fig. 3:**
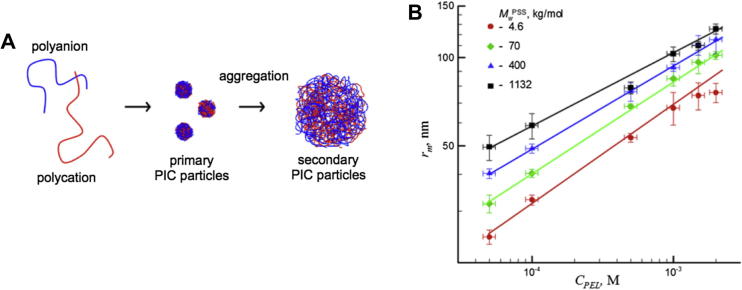
(A) Schematic presentation of the processes occurring during mixing of polyelectrolytes and formation of PIC particles. (B) PIC particle size (r_m_) dependence on polyelectrolyte concentration (C_PEL_) using different PSS molecular weight with PDADMA. n−/n+ = 1.5. Adapted with permission from Ref. [44].

Recent work by Starchenco and co-workers rationalised the mechanism of PIC particle self-assembly as the sequential nucleation of polyions as primary molecular complexes, which then aggregate into colloidal complexes (Fig. 3A) [44]. The authors explained this behaviour by the increase in the Debye length of the nanoparticles as they become bigger, thus creating more intense repulsive electrostatics between the “secondary” complexes. In this article, the size of PIC particles from PSS/PDADMA was studied as a function of polyelectrolyte concentration. As shown in Fig. 3B, a linear correlation was found between polymer concentration and complex size, regardless of the molecular weight of PSS used (4.6–1132 KDa). It was observed that, at the lowest concentration of polyions tested (50 μM), particles with a radius of 23 and 50 nm were found when using the lowest and highest PSS molecular weight, respectively. As observed here, the size of PIC particles can be finely tuned by a combination of polyion length and concentration.

Often during the assembly of PIC particles, high concentrations of the starting polyelectrolytes lead to the formation of micron-sized complexes with poor colloidal stability [28], [33]. As a consequence, it can be generalised that dispersed PIC nanoparticles can only form in polyelectrolyte mixtures below a critical concentration, above which the system flocculates or precipitates (ca*.* <1 mg/mL) [3]. The concentration restraints during PIC particle preparation can be a limitation when it is required to prepare concentrated dispersions of these complexes. Nevertheless, Müller et al. showed that it was possible to centrifuge and redisperse PIC particles from PDADMA and poly(maleic acid-*alt*-α-methylstyrene), and that consecutive centrifugation cycles allowed to refine the polydispersity of the complexes, ultimately achieving monomodal size distributions without affecting their initial size during the process [45]. Ouyang et al. found the same effect on PSS/PDADMA complexes of 318 nm in diameter and polydispersity index (PDI) = 0.38 [46]. After one centrifugation, the size of these complexes dropped to 250 nm, showing a much narrower size distribution (PDI = 0.06). After a second centrifugation, particle size was maintained (251 nm) and the PDI could be narrowed further down to 0.04. As such, it is possible to obtain concentrated PIC particles dispersions by centrifugation, although the final morphology of the complexes may be affected.

### 2.3 pH

**Fig. 4:**
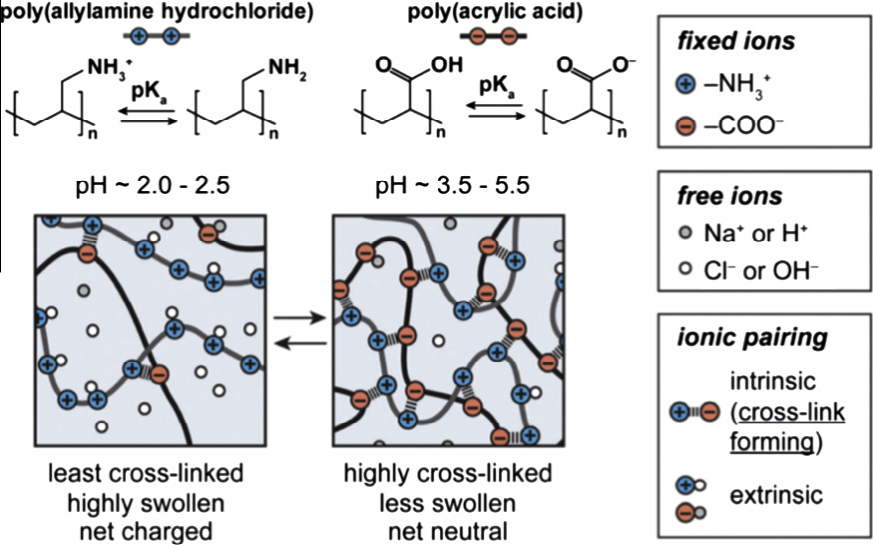
Illustration of the pH-dependent binding between poly(allylamine hydrochloride) and poly(acrylic acid). Adapted from Ref. [48].

From a biomedical perspective, polyelectrolytes can be classified as either weak or strong whether they undergo partial or full ionisation at physiological pH, respectively [35]. As a result, as opposed to strong polyelectrolytes, the charge density of weak polyions will strongly depend on the pH found in different tissues and organelles (4.5–7.4) [47]. Variations within this pH range can lead to significant changes in polyelectrolyte binding and PIC particle stability from the loss or gain of valency (*i.e.* charges per molecule). A good example of this pH-responsive behaviour of polyion complexes was reported by Han et al., where the authors studied a system comprised of a strong poly(allylamine) (PAH) and weak poly(acrylic acid) (PAA) polyelectrolytes at different pHs (Fig. 4) [48]. At low pH (2.0–2.5), PAA is only deprotonated to a small extent so that more swollen and loose complexes are formed as a consequence of its low charge density. However, at higher pH (3.5–5.5) the PAA chains are more ionised, and thus more packed complexes form with PAH from an increase in the number of electrostatic cross-links.

**Fig. 5:**
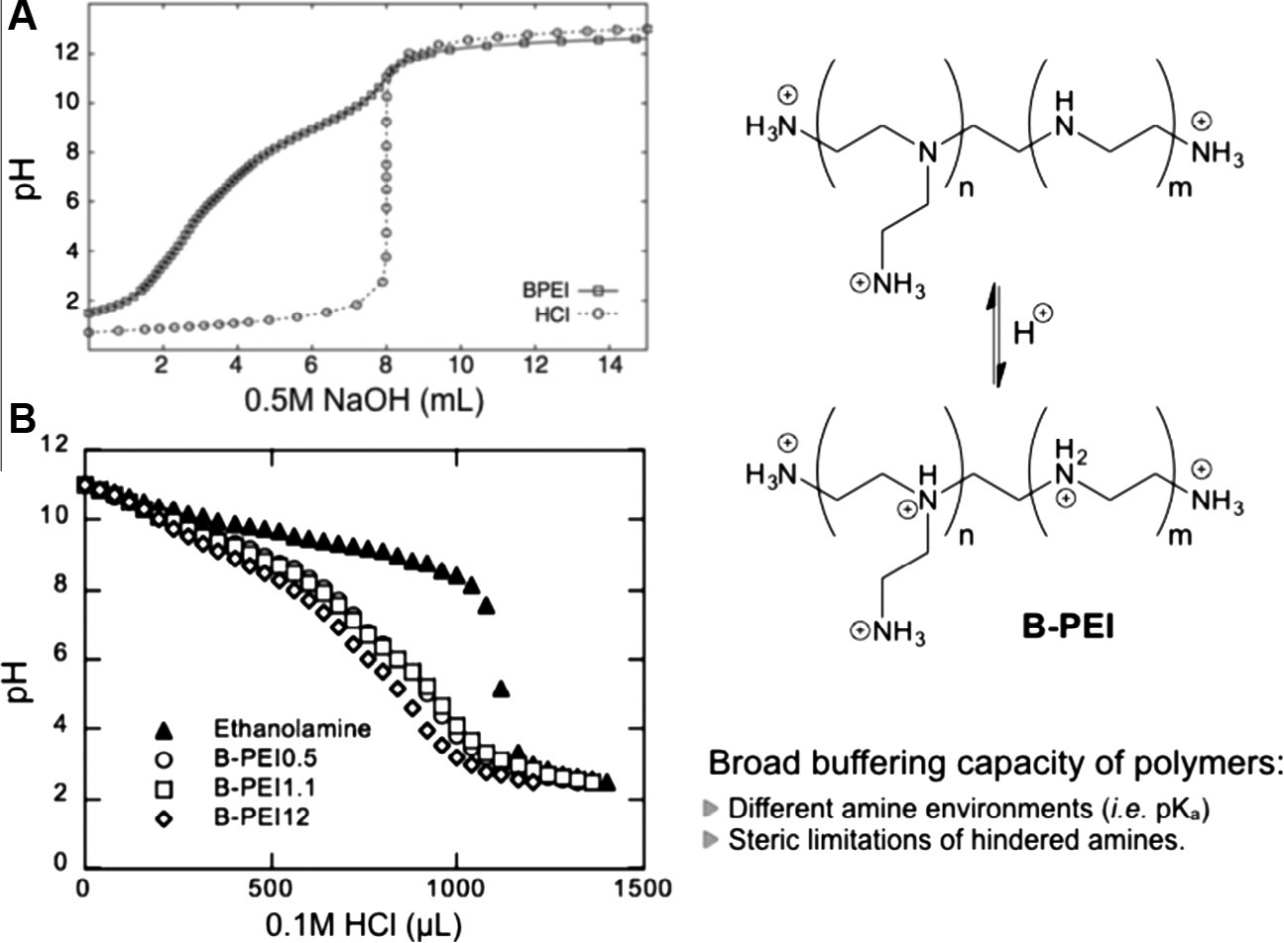
Buffering capacity of branched poly(ethylene imine) (B-PEI). (A) Titration of 25 KDa B-PEI acidified with HCl (also titrated in the same concentration as control). (B) Potentiometric titration of 0.5, 1.1 and 12 KDa B-PEIs, compared to that of ethanolamine as control for a small monofunctional amine. Adapted from Refs. [49], [50].

Weak polyions can therefore change their (de)protonation degree and charge density with changes in biological pH. In addition, the different chemical environments and sterics result in a range of different pK_a_ values for the same monomer in different positions of the polyelectrolyte. This is specially the case for branched polymers and translates into broad buffering capacity. As such, the changes in pH found in different physiological settings have been exploited to trigger the release of drugs and DNA from PIC particles by changes in the ionisation of the complexing polyions. A classic example of this behaviour is the so-called ‘proton sponge’ effect displayed by PEI, which allows polyplexes to escape the acidic endosome after cellular uptake (Section 3). For example, branched PEI (B-PEI) displays a wide buffering window from approximately pH 2–10 regardless of its molecular weight (Fig. 5) [49], [50], and can therefore buffer the influx of protons into the endosome, ultimately causing an increase in osmotic pressure that causes the organelle to burst and release the polyplex into the cytosol.

Other relevant examples of pH-responsive PIC particles include enzyme [51] and antimicrobial [52] delivery, which are based on nanoparticle disassembly or swelling upon polyion (de)protonation, respectively. As shown by Sui et al., reversible pH-responsive PIC particles can be prepared by blending a weak polyacid (PAA) with a strong polybase (PDADMA) into PDADMA-*co*-PAA [53]. Mixing this copolymer with PSS at pH 4.1 led to the formation of PIC particles, which dissolved at pH > 5.7 due to the deprotonation of PAA in the copolymer and subsequent repulsion between PSS and this partially anionic copolymer. When the solution was acidified again, PIC particles reformed at pH 4.5–5.5, thus demonstrating the reversibility of the process.

**Fig. 6:**
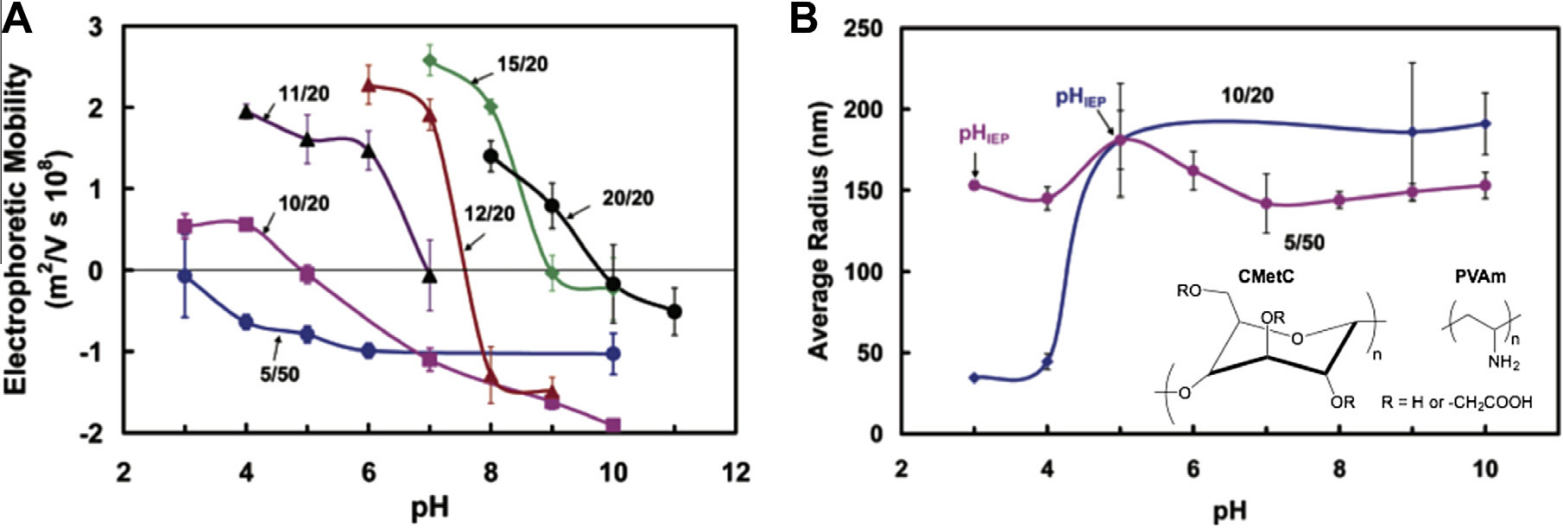
Effect of pH during preparation of PIC particles from carboxymethyl cellulose (CMetC) and poly(vinylamine) (PVAm) at different mixing ratios (labels: CMetC/PVAm) on their charge (A) and size (B). Adapted from Ref. [22].

Similarly, the pH chosen to self-assemble two polyions can affect their charge densities and therefore the final characteristics of the resulting nanoparticles. Feng et al. studied the effect of pH during PIC particle preparation from carboxymethyl cellulose (CMetC) and poly(vinylamine) (PVAm) in different proportions (*i.e.* mixing ratios) [22]. The authors observed a mixing ratio-dependent effect that originated more positive or negative PIC particles from more acidic or basic pH, respectively (Fig. 6A). This result is consistent with the higher (de)protonation of polyions at higher pH values. However, lower CMetC (weaker polyion) contents led to less pronounced changes in PIC particle charge, as expected from the higher pH-sensitivity of CMetC over PVAm. Size-wise, although an increase in one unit of pH led to a 4-fold increase in size at a 10/20 ratio (CMetC/PVAm), PIC particle size was not very sensitive to changes in pH above their isoelectric points (Fig. 6B).

### 2.4 Ionic strength

The addition of small electrolytes can aid the self-assembly of PIC particles by shielding the intramolecular charge repulsions of polyions, thus increasing the flexibility of the polymers and their ability to self-assemble [54]. On the other hand, high salt concentrations can lead to strong electrostatic screening between polyelectrolytes and quench the nucleation of PIC particles [3]. Therefore, the ionic strength of the medium must be carefully selected to successfully assemble PIC particles.

**Fig. 7:**
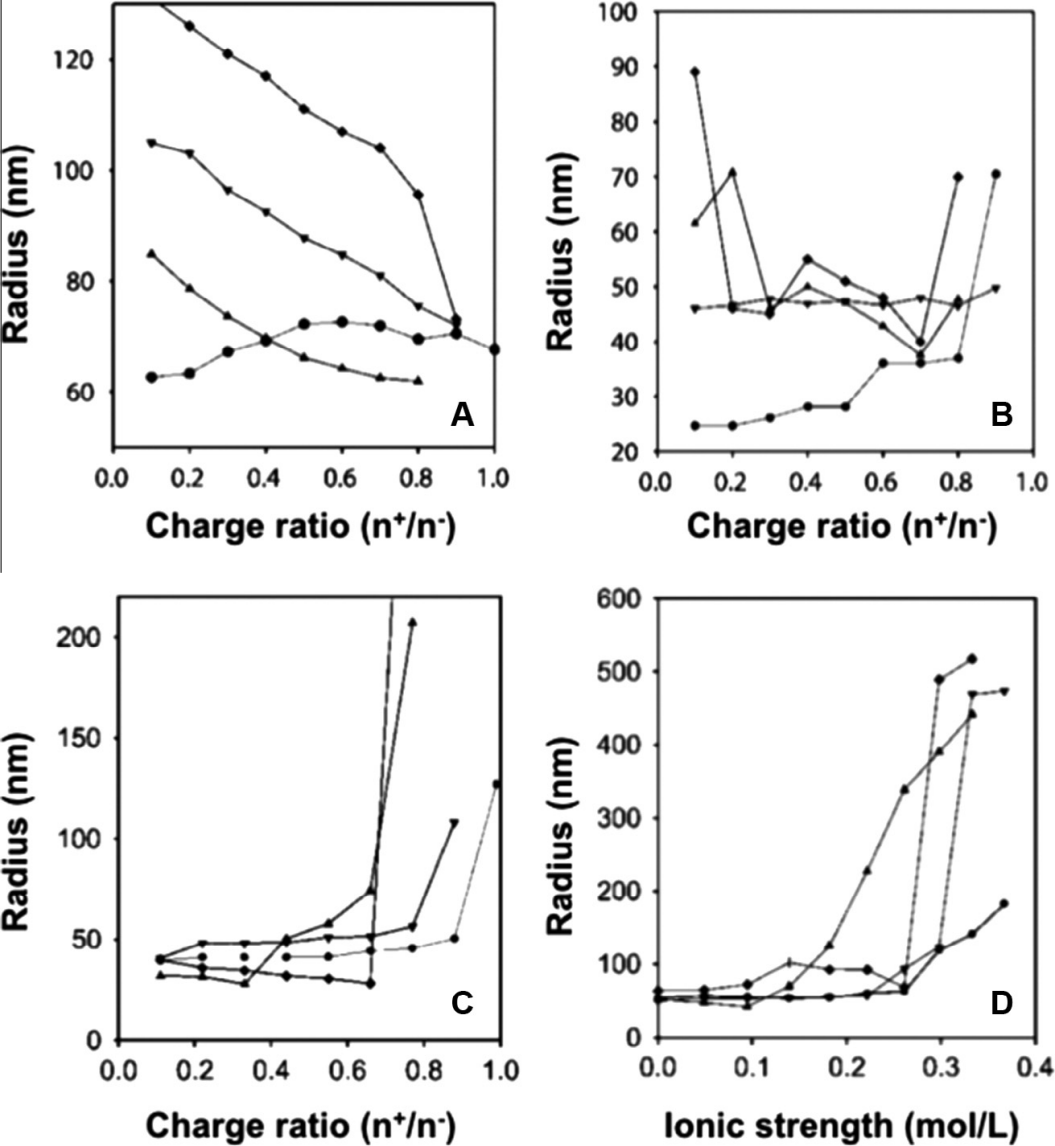
Size of PIC particles prepared from PSS and p(DADMA)-co-(NMVA) with 100% (●), 75% (▾), 53% (▴) and 24% (♦) of PDADMA (*i.e.* cationic) content in pure water (A), 10 mM (B) and 100 mM (C) NaCl. (D) Swelling of PIC particles prepared in water at increasing NaCl concentrations. Adapted from Ref. [21].

To this end, Dautzenberg et al. studied the effect of NaCl during the assembly of PIC particles from PSS and p(DADMA)-co-(NMVA) copolymers (Fig. 7A–C) [21]. In general, the sizes of PIC particles decreased when the polyions were mixed in the presence of higher concentrations of NaCl, probably due to a better packing of more flexible polyelectrolytes. The difference in sizes found between copolymers in water decreased in 10 mM NaCl, ultimately forming particles of the same size at 100 mM NaCl. Overall, polyions with stronger charge densities (*i.e.* lower NMVA content) were more tolerant to NaCl during PIC particle preparation. The authors also observed that particles prepared in pure water swelled from 100 nm up to 1 μm in diameter when exposed to NaCl once assembled (Fig. 7D) [21]. This post-assembly effect of NaCl is due to its infiltration within the core of the complexes, thus neutralising the electrostatic cross-links between polyelectrolytes and subsequently causing pre-formed complexes to swell. In later work, the same group studied the kinetics and mechanism of salt-induced PIC particle swelling by static light scattering [19]. The authors observed that the increase in size occurs quickly after the addition of NaCl (ca*.* < 10 min) as a combination of swelling and subsequent aggregation of swollen complexes. Once again, copolymers with higher charge density (*i.e.* PDADMA content) showed improved tolerance to the swelling effect caused by NaCl due to their higher avidity, requiring in some cases twice the concentration of NaCl to swell compared to less charged copolymers (Fig. 7D).

Wang et al. studied the effect of increasing concentrations of KBr on complexes made from PDADMA and PSS [55]. The authors found dense PIC particles in the absence of KBr, which started swelling with increasing amounts of KBr (doping) due to electrostatic shielding. Higher KBr concentrations (ca*.* 1.5 M) led to disperse colloidal PIC particles, which dissolved above 1.8 M KBr. It is noteworthy that the dilution of concentrated KBr samples with water re-assembled PIC particles, evidencing the reversible nature of salt doping effects. Also the high molecular weights of the polymers used in this study (200 KDa PSS and 400 KDa PDADMA) required higher concentrations of KBr to shield such multivalent interactions and trigger these phase transitions. It is expected that PIC particles made from shorter polyelectrolytes with weaker avidities would require lower doping levels to undergo similar structural changes: *e.g.* particles in Fig. 7D from 66 KDa PSS and 100 KDa PDADMA required only about 0.35 M NaCl to swell twice in size.

Physiological electrolytes may also cause swelling and compromise the functionality and stability of PIC particles in biological settings. If salt-tolerant particles are required, the assembled chains of polyelectrolytes can be covalently cross-linked to maintain the complex together even after the weaker electrostatic forces have been screened with salt [56], [57]. Nevertheless, the swelling of PIC particles with small electrolytes has been exploited to release entrapped drugs from within the complex as a result of its increased permeability [31], [32].

### 2.5 Mixing ratio

This is the proportion of positive and negative charges in the polyelectrolytes that are mixed. Mixing ratio not only affects the final size and charge of PIC particles, but also their biological activity. It must be pointed out that, as seen in the previous section, the degree of ionisation of polyelectrolytes is pH-dependent, and therefore the ‘effective’ mixing ratio will vary for the same mixture of polyions at different pH values [6]. Mixing ratios are denoted regardless of the pH by the fraction or percentage of acidic and basic monomers in solution (*e.g.* ‘N:P’ ratios are normally used in polyplex formulations to indicate the ‘N’ number of amine monomers complexed with ‘P’ phosphate groups in the nucleic acid).

Non-stoichiometric mixing ratios are necessary to give colloidal PIC particles a stabilising shell of the polyion in excess (Fig. 1). On this basis, and as long as other variables such as pH, ionic strength and the polyions structure are kept constant, the absolute value and variations in ζ potential of PIC particles can be predicted from changes in their mixing ratio.

Given the strong dependency of numerous PIC particle systems on mixing ratio, it is always necessary to assess a range of ratios in order to draw consistent and representative conclusions of PIC particle behaviour. The range of mixing ratios usually evaluated varies from very positive-rich mixtures in polyplexes (up to 50 in N:P) [58] whereas other systems just explore values closer to equimolarity (*e.g.* 0.2–1.8 [59] or 0.5–2.0 [4] in n−/n+).

**Fig. 8:**
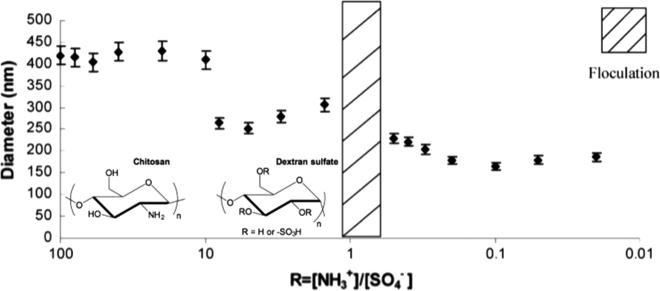
Average hydrodynamic diameter of PIC particles from chitosan and dextran sulphate at different [NH_3_^+^]/[SO_4_^−^] ratios. Adapted from Ref. [54].

A thorough mixing ratio study was conducted by Drogoz et al., who studied the formation of PIC particles from mixtures of chitosan and dextran sulphate two orders magnitude above and below equimolarity [54]. Firstly, the authors observed that the absolute charge of the complex was that of the polyion in excess. Secondly, the size of the nanoparticles was distributed in three ranges of [NH_3_^+^]/[SO_4_^−^] ratios: above 10 (420 nm), between 10 and 1 (250 nm) and below 1 (200 nm) (Fig. 8). The authors explained these three particle sizes from the different nucleation mechanisms taking place in excess of either polymer. At mixing ratios close to neutrality, unstable complexes formed and flocculated. The authors also found that at high excess of either polyion (5-fold and above), more than 70% of this polymer stays uncomplexed free in solution. Comparing this to other examples in the literature, whereas mixing ratio has the same effect on the ζ potential for other PIC particles, its influence in size is not consistent and seems to be influenced by other variables (*e.g.* molecular weight and strength of the polyions) [56], [60], [61].

### 2.6 Mixing order

The order in which polyelectrolytes are mixed together will dictate what polyion is in excess at the beginning of the assembling process. It is very important to notice that for the preparation of non-stoichiometric PIC particles, for example at [n+/n−] = 2, if the polyanion is added to the polycation there is always an excess of positive charge in the complexes, and repulsive forces between them help maintain colloidal stability. However, if the same polymer mixture is prepared by addition of polycation (in excess) to polyanion, the negative charge of the initial coacervates will reach a neutral value half way through the addition of polycation. As a result, neutral particles can flocculate if polyelectrolytes are mixed in the wrong order. Therefore, it is suggested that the polyion in deficit is added to the one in excess, which results in different nucleation dynamics depending on the nature of the polyelectrolytes [54].

The effects of the order of addition become evident when titrating mixing ratios of polyelectrolytes. For example, Dautzenberg et al. observed that the mixing order of polyelectrolytes not only affected the size of the resulting nanoparticles, causing up to a 2-fold change in size, but also the stability of these complexes to NaCl, probably due to a different packing of the polymers [21]. Other studies in the literature have shown rather dissimilar results, finding either a strong dependency or almost negligible effect on the final morphology of the complexes [3], [62]. Discrepancies between studies should be attributed to the effects of other variables (*e.g.* molecular weight and polyion strength), and therefore each case should be evaluated individually.

### 2.7 PIC particle cross-linking

PIC nanoparticles, in particular those prepared from low molecular weight polymers, partially ionised polyions or when exposed to high salt concentrations, are in equilibrium with free polyions in solution [28]. This equilibrium can be displaced towards the release of polyions from the nanoparticles in circumstances that weaken their electrostatic binding (Fig. 1). As a result, PIC particles can disassemble when diluted or exposed to other charged species [29].

Alternatively, the stability of PIC particles can be greatly enhanced by covalently cross-linking the complexed polyions, thus preventing the detachment of polymer chains from the nanoparticle. In essence, cross-linking works by ‘polymerising’ polyelectrolyte chains, thus dramatically increasing the overall molecular weight of the polymer and consequently its binding affinity (see Section 2.1.). To this end, polyelectrolytes can be cross-linked during or after particle self-assembly, depending on whether the polyelectrolytes carry cross-linking groups in their structure or if a third component that cross-links pre-assembled complexes is added to the system, respectively.

An illustrative example of PIC particle post-cross-linking was reported by Hsieh et al., who prepared nanoparticles from poly(acrylic acid) (PAA) and poly(l-lysine) (PLL) cross-linked at their core and/or shell [56]. The amines or carboxylic acids found in the shell of these PIC particles were cross-linked using genipin or cystamine, respectively. PLL chains in the core were cross-linked by silica deposition using orthosilicic acid as precursor. These cross-linked particles tolerated up to a 150-fold dilution.

The post-assembly cross-linking of PIC particles has the advantage of not requiring the functionalisation of the polymers with cross-linking groups, thus being applicable to virtually any polyelectrolyte with the suitable functional group (*e.g.* amines or carboxylic acids in the case described). Given the wide application of polyamines in gene delivery, other amine cross-linkers have been applied to polyplex preparation, including glutaraldehyde [63], citric acid [64], carboximidates and *N*-hydroxysuccinimide esters [65].

**Fig. 9:**
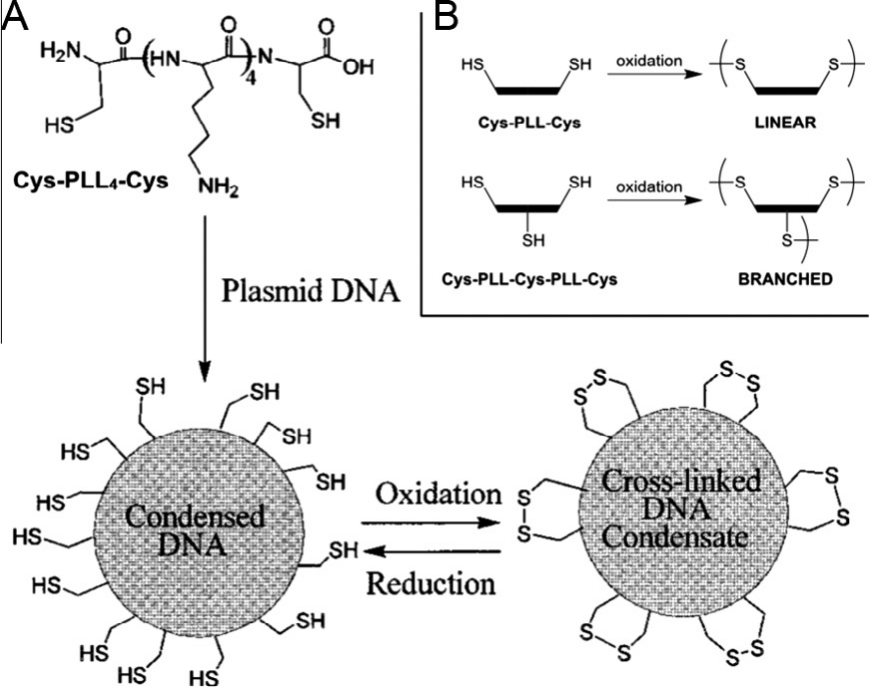
(A) Formation of cysteine-containing PLL/plasmid DNA nanoparticles. The thiol groups in cysteine (Cys) residues can cross-link PLL molecules through oxidation into disulphide bridges. These cross-links will break under reducing conditions (*e.g.* human cytosol), destabilising the nanoparticles and thus allowing the release of DNA. (B) Schematic representation of the linear and branched cross-linking of these polyplexes that forms upon oxidation of PLL containing two and three Cys residues, respectively. Adapted from Ref. [66].

Alternatively, functionalised polyelectrolytes allow to cross-link the PIC particles as they are formed. By far, the most used group to functionalise and cross-link the polyions in PIC particles are thiols, given their mild reaction conditions and reversible cross-link into disulphide bridges. For example, McKenzie et al. described the preparation of disulphide cross-linked polyplexes from plasmid DNA and PLL decorated with cysteine residues [66]. Polyplexes prepared from a difunctional Cys-PLL-Cys (Fig. 9A) did not show an increase in stability compared to a control PLL of the same molecular weight. However, when three cysteines were incorporated to PLL (Cys-PLL-Cys-PLL-Cys), the resulting nanoparticles tolerated 2.5 times the maximum concentration of salts Cys-PLL-Cys polyplexes could stand. The effect of this extra cysteine can be explained from the cross-linking of PLL into branched structures (Fig. 9B), leading to a higher charge density as discussed in Section 2.1. However, a dramatic increase in particle size was observed for Cys-PLL-Cys-PLL-Cys polyplexes (1.4 μm) compared to the analogous Cys-PLL-Cys (52 nm), an effect the authors attributed to inter-particle cross-linking by the former polyion. When exposed to reducing conditions, the disulphide cross-links in these polyplexes broke, leading to a sudden loss in the PLL’s apparent molecular weight and thus triggering the disassembly of the nanoparticle and DNA release (Fig. 9A).

As described, cross-linking short polyions through labile cross-linkers allows the preparation of stimuli-responsive PIC particles, which can breakdown in the presence of agents that degrade such labile linkers, thus causing a sudden loss of charge density and binding affinity between polyions. As so, the reducing conditions found in the cytosol have been exploited to trigger intracellular drug and nucleic acid delivery through the reduction of disulphide cross-linked vehicles [67].

**Fig. 10:**
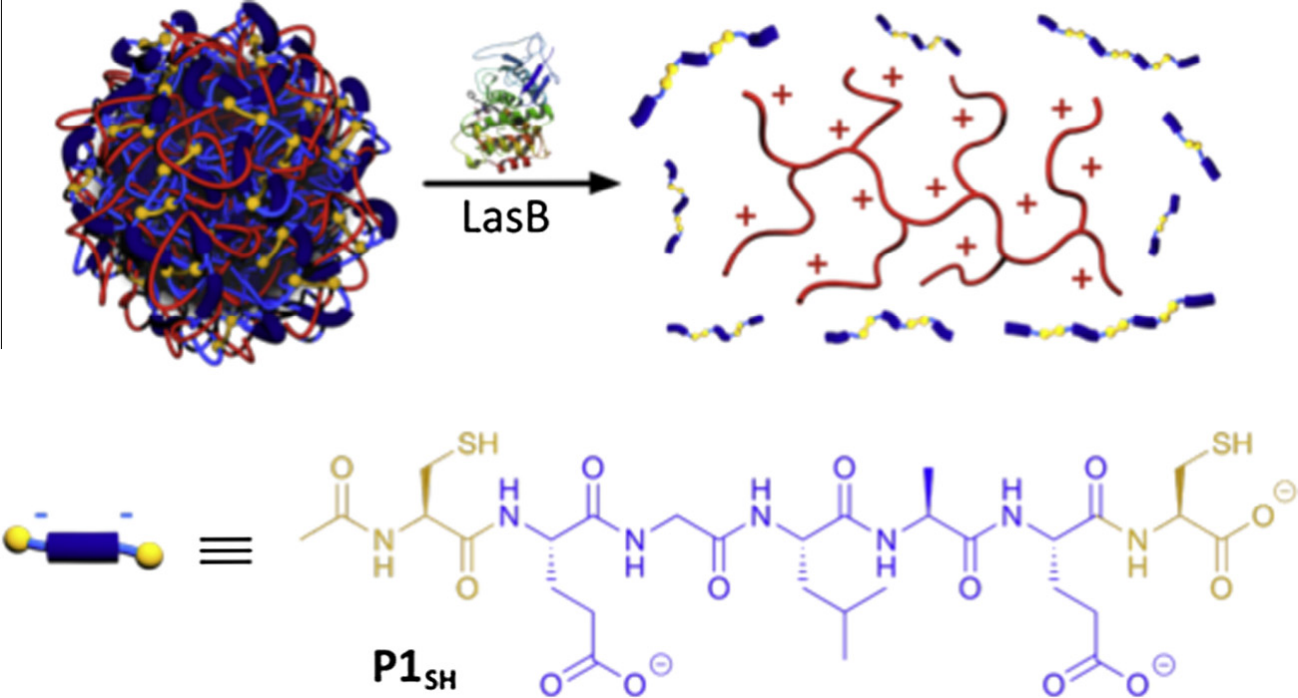
LasB-triggered disassembly of responsive PIC particles prepared from branched PEI (red polycation) and anionic peptide **P1_SH_**. The cysteine residues allowed peptide cross-link (in yellow) and the -GLA- amino acid sequence in the peptide (dark blue) hydrolysed by LasB. Adapted from Ref. [68]. (For interpretation of the references to colour in this figure legend, the reader is referred to the web version of this article.)

Our group combined two different labile linkers to develop dual-stimuli-responsive PIC particles, which disassembled in the presence of enzymes and reducing agents [68]. In this system, PEI was complexed with a short anionic peptide bearing two cysteine residues to allow their oxidative cross-linking (**P1_SH_**, Fig. 10), endowing PIC particles with redox-responsive properties like those in the previous example. In addition, the amino acid sequence in those peptides could be specifically degraded by a bacterial elastase (LasB), thus providing another point to cleave the cross-linked peptide chains and ultimately the PIC particles themselves.

As just illustrated, the integrity of the cross-links in such particles is of paramount importance to ensure particle stability, but at the same time, labile cross-links can be designed as sacrificial groups that trigger particle disassembly and release of their cargo when cleaved.

## 3 Application of PIC particles in gene therapy

**Fig. 11:**
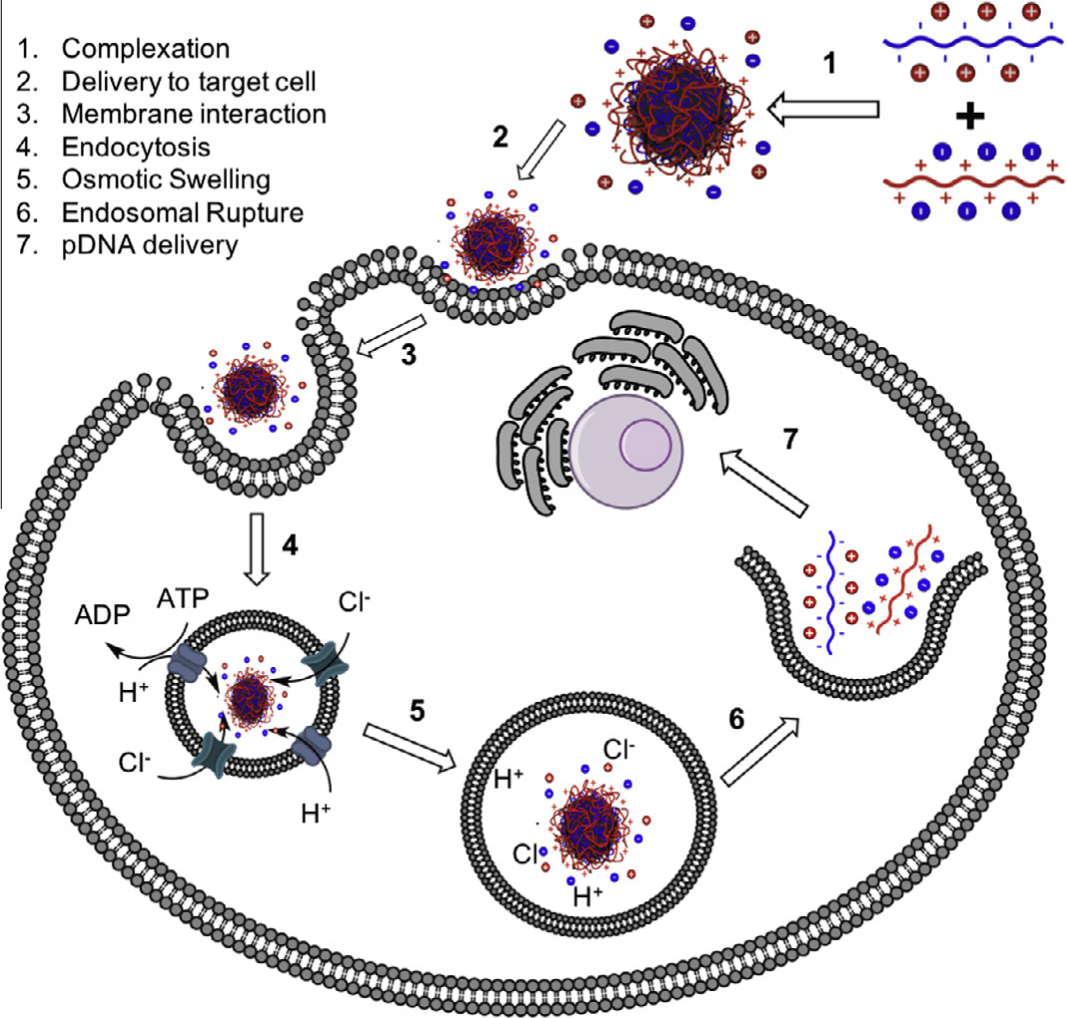
Schematic representation of the uptake of polyplexes into cells, illustrating the different challenges faced in the development of PIC particles for gene delivery.

Possibly the widest application of PIC particles in biomedicine has been the non-viral delivery of nucleic acids [69], [70], [71]. Nucleic acids are strong polyelectrolytes that present one negative charge per nucleotide at neutral pH. This high negative charge density in nucleic acids poses a big challenge when trying to deliver them across negatively charged cellular and nuclear membranes to enable gene therapy (Fig. 11). Moreover, foreign nucleic acids will be readily degraded in biological environments, thus compromising their half-life *in vitro* and *in vivo*. In natural systems, this is often compensated by protecting the nucleic acids by complexation with positively charged proteins, such as histones [72], or within viral capsids [73]. Not surprisingly, the gene delivery community has placed its attention into mimicking nature’s strategy using positively charged polyelectrolytes [69], [70], [71].

Often, it has been argued that PIC particles made with nucleic acids and cationic polyelectrolytes, *a.k.a.* polyplexes, can address some of the limitations observed in viral gene delivery (*i.e.* immunogenicity and scalability). However, as will be explained in the following sections, synthetic polyplexes developed so far for non-viral delivery have been found to have separate issues on their own that need to be overcome including stability, toxicity, cellular uptake, endosomal escape and for nucleic acids, delivery to the nuclei.

### 3.1 Toxicity

Most of the polymers employed in non-viral gene delivery are strong cationic polyelectrolytes, such as PEI, PLL or chitosan. However, it has been long established that most cationic polymers are cytotoxic and cause damage to cells [74], [75]. The mechanism for this toxicity though is still widely debated. PEI and other polycations can cause disruption of lipidic bilayers. This is thought to be driven by electrostatic (and possibly hydrophobic) interactions between the negatively charged lipidic membrane and these polyelectrolytes, which often results in depolarisation of the membrane and the formation of pores [76]. This is further evidenced by the release of cytosolic contents such as lactate dehydrogenase [77].

Yet it is also postulated that cationic polyelectrolytes (*i.e.* PEI) can trigger cell apoptosis [78] by release of cytochrome *c* from mitochondria upon intracellular interaction of the polymer with organelles. A similar interaction may be expected here where interaction of PEI with the anionic organelle membrane leads to pore formation and release of the organelle contents [79]. Even if these two mechanisms are working in isolation or cooperatively towards cell death, this toxicity does need to be considered in the future development of PIC particles for gene delivery.

Several strategies have been reported to minimise the toxicity of these polyplexes. For instance, Zhao et al. found that inclusion of chitosan in a PEI/DNA complex significantly reduced the toxicity when compared to the PEI/DNA complex alone [58]. The authors argued that this decrease in cytotoxicity was related to the lower toxicity of chitosan, due to its increased steric hindrance and charge shielding ability.

The simplest way to reduce toxicity of cationic polymers though is reducing the molecular weight of the polyelectrolyte used [74], [80]. In depth studies have demonstrated that both PEI and PLL have lower toxicity when the molecular weight is reduced [77], [80]. However, as mentioned in the previous section, reducing the molecular weight of the polyelectrolytes has a dramatic effect on the stability of the PIC particles formed. With gene delivery, this is often translated into a lower transfection efficiency [81]. As such, much work has focused in the application of cross-linkers that can reversibly increase the polyelectrolyte molecular weight (Section 2.7) [82].

Disulphide formation has often been the preferred method of cross-linking. Of relevance to the delivery of nucleic acids is the ability of these cross-linkers to be cleaved in the presence of reducing media. This is what happens upon entry into the cell, where the concentration of glutathione is increased up to 100-fold when compared to the extracellular environment [83]. Using this strategy, transfection efficiencies with cross-linked 1.8 KDa PEI are equivalent to that of PIC particles made with 25 KDa PEI [84].

**Fig. 12:**
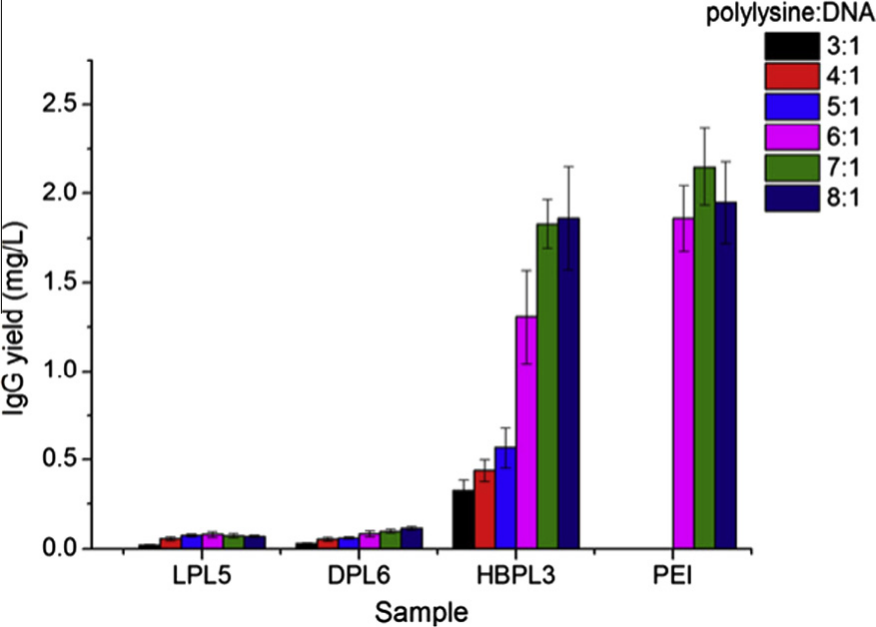
Comparison of the percentage of eGFP positive cells obtained after 24 h transfection with polylysine analogues of comparable molecular weight. Reproduced from [87].

It was thought until recently that dendritic polymers could have reduced toxicity compared to linear analogues [85], [86]. However a comprehensive study had not been completed on a comparison of the toxicities of linear vs. dendritic PLL. Klok et al. found that dendritic equivalents of PLL and hyper-branched versions have improved transfection efficiencies compared to their linear analogues (Fig. 12) [87]. However, when carrying out a toxicity study it was found that dendritic and hyper-branched PLL has greater cytotoxicity compared to an equivalently sized linear analogue [88]. The key factors that were highlighted for this increase in cytotoxicity were osmotic shock and cell membrane damage. Osmotic shock causes initial disruption to cells which leads to a marked decrease in EC_50_ values for dendritic and hyper-branched structures. Whilst the membrane disruption characteristics show that the dendritic and hyper-branched structures also cause cell apoptosis due to their interaction with mitochondria which leads to the release of cytochrome *c* as discussed previously [78]. What is interesting to note for the studies by Kadlecova is that although there is increased cytotoxicity seen for both the dendritic and hyper-branched forms of PLL, the hyper-branched structure is much simpler in its synthesis however, and has almost identical effects in terms of toxicity and transfection.

Finally, it is worth mentioning that conjugation of PEG to one of the polyelectrolytes is another strategy to minimise toxicity of the polyplexes. This strategy results in the formation of PIC micelles [12], [13], which are stabilised by a PEGylated corona. It is thought that the reduction in toxicity is achieved thanks to the formation of a hydration shell around the polymer reducing interactions with the membrane during delivery [89], [90]. This strategy has been used successfully, for example when coupled to PEI, to reduce the toxicity of the polyplexes used for *in vitro* delivery studies [91], [92]. Further examples can be found in specialised reviews [12], [13], [93].

### 3.2 Transfection efficiency

The other key factor to consider when developing a non-viral gene delivery vehicle is transfection efficiency. How efficiently a gene is expressed (or knocked-down for small interfering RNA) is normally a consequence of several factors, including circulation half-life, uptake, endosomal escape and transfer to the nuclei in the case of plasmid DNA [94], [95]. Representative examples of the effect of these factors over transfection efficiency will be described in the next sections.

#### 3.2.1 Circulation half-life

As mentioned earlier, nucleic acids when introduced in biological environment (*i.e.* the body or serum) have a very poor circulation half-life. This is because nucleic acids are quickly degraded by extracellular nucleases, which are responsible for the elimination of any foreign genetic material [96]. Complexation of these nucleic acids in polyplexes is thus the first step to improve their circulation half-life. Normally, polycations are added in excess and nucleic acids are therefore contained within the neutral core, protected this way by the cationic corona from the action of these nucleases.

However, as described in the first section, the stability of the formed polyplexes would strongly depend on several factors. Assuming that parameters such as concentration, pH and ionic strength can be maintained constant when comparing different polyelectrolytes in the same application, factors such as polyelectrolyte molecular weight and topology dictate the stability of the formed polyplexes. This is often the case when comparing pDNA (normally over one thousand base pairs) to oligonucleotides such as siRNA, which have a significantly smaller size (20–25 base pairs). This was nicely illustrated by Seymour et al. with their reducible polycation-based system [97]. While a weight ratio of 5:1 (N:P 1.9:1) was enough to achieve complete retention of pDNA in an agarose gel, weight ratios of up to 20 (N:P 7.5:1) were insufficient to fully conjugate all of the siRNA employed. As expected, polyplex formation in the absence of other salts, resulted in full conjugation of the siRNA with the 20:1 weight ratios, highlighting once again the role of ionic strength in the formation and stability of PIC particles.

Another explanation for the reduced half-life of polyplexes is opsonisation, whereby soluble proteins (opsonins) bind to the surface of complexes leading to their targeted destruction through phagocytosis and subsequent removal from circulation [98]. This process is thought to occur due to the non-specific binding of serum proteins to the positively charged polymer [99].

**Fig. 13:**
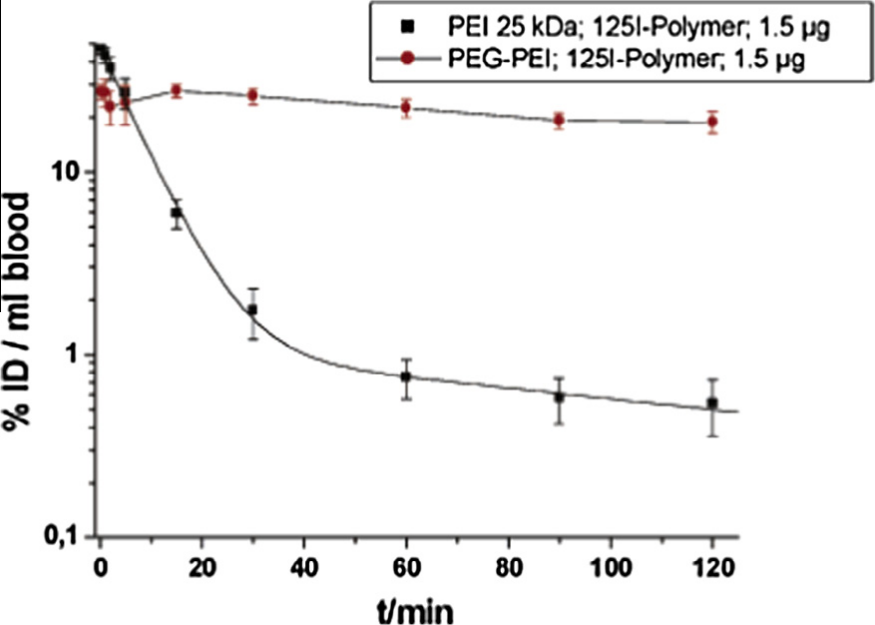
Pharmacokinetic study showing the effect of PEGylation on circulation half-life as measured by radioactive isotopes ^125^I. Reproduced from [101].

It is commonly understood that circulation half-life of drugs in the body can be improved through the attachment of hydrophilic residues [100]. Again, in gene delivery this is often achieved through the introduction of PEG residues within the polymeric structure [12], [13], [89], [93]. Kissel et al. evaluated the effect of PEGylation on the pharmacokinetic study carried *in vivo* of PEI polyplexes [101]. Less than 1%/mL of the injected dose (ID) was detected after 2 hours, following injection of polyplexes containing 1.5 μg of pDNA. However, over 20% was still in blood when these polyplexes were PEGylated (Fig. 13).

#### 3.2.2 Uptake

**Fig. 14:**
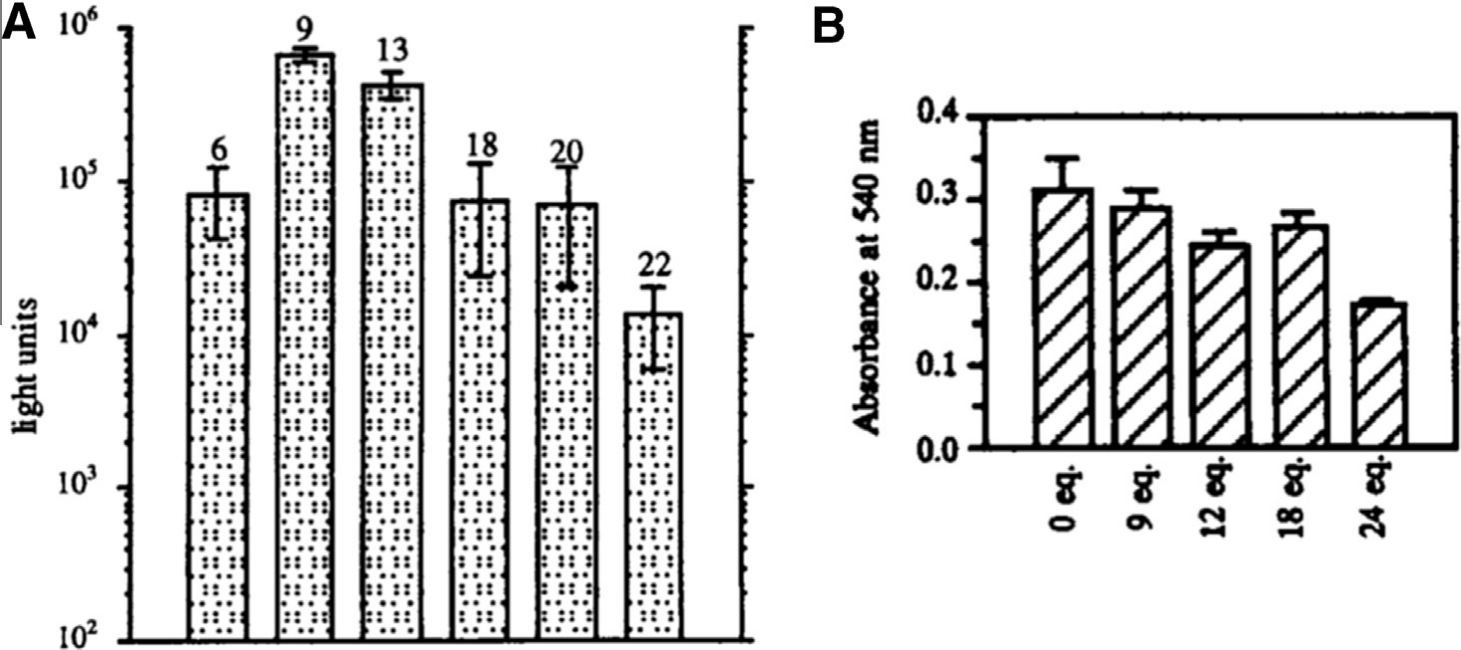
Transfection efficiency (A) of PEI/pDNA increases with increasing equivalents of PEI, and has its maximum at 9–13.5 equivalents when toxicity towards 3T3 cells (B) starts to be apparent. Reproduced from [102].

To be transfected, polyplexes have to be efficiently taken up by the cell. This is normally facilitated by the excess of positive charge in the corona of the polyplex. This has been demonstrated for instance using PEI [102], where transfection efficiency was increased with increasing amount of PEI (*i.e.* N/P ratios) (Fig. 14). However, increasing PEI content in the PIC particle also resulted in an increase in cytotoxicity, and thus transfection efficiency eventually was compromised.

Alternatively targeting ligands, that are recognised by the cell surface thus promoting binding and endocytosis of the polyplexes, can be employed to improve transfection efficiency. For instance, Behr and co-workers demonstrated that the use of glycosylated or RGD PEI derivatives, resulted in higher transfection efficiencies when compared to polyplexes prepared with “pristine” PEI [103]. Typically, transfection efficiencies increased by 1–2 orders of magnitude for the targeted PIC particles. Other targeting ligands such as antibodies, folic acid or prostaglandins have been [104] or could be employed [105] in the development of targeted polyplexes.

#### 3.2.3 Endosomal escape

One of the key barriers that polyplexes need to overcome to mediate efficient transfection is the endosome. Uptake normally results in polyplexes trapped within the endosome, where the cell will try to either digest them or recycle the contents [106]. However, nucleic acids need to be transported into the cytosol, where they can carry out their function (*e.g.* siRNA) or be further trafficked into the nuclei (pDNA).

Part of the reason why PEI has garnered more interest than other polycations is because of its inherent ability to act as a buffer within the endosome (Fig. 5), causing osmotic swelling and potential leakage of the endosome contents (‘The proton sponge effect’) [107]. Behr et al. hypothesised that the reason for endosomal rupture is the protonation of the amine groups in PEI which subsequently leads to an influx of Cl^−^ counterions and water. It is known that protons can be pumped against the electrochemical gradient that is formed due to the presence of ATPase V type pumps, which helps support this hypothesis [108]. This influx then leads to osmotic swelling, eventually rupturing the endosome.

However, there is still debate as to whether this is the method for endosomal release. Benjaminsen et al. recently found that PEI complexes reach lysosomes to a high extent, but do not cause a change in lysosomal pH [49]. Instead Benjaminsen states that the failure of the ‘proton sponge model’ is that the swelling caused by this process would not cause rupture. This opinion directly contradicts computational calculations that have been made which support the proton sponge effect [109], [110]. Here it has been modelled that osmotic swelling is highly dependent on polymer pK_a_ as protonation needs to be caused by entry into the endosome.

In line with Benjaminsen’s observations, some have argued that the sponge effect should result in an increase in pH [111]. However this doesn’t have to be the case because if enough ATP is present in the cytosol, the lysosome could counteract PEI buffering with the influx of more protons. Even if the pH is not changed, osmotic swelling should occur upon the recruitment of protons to neutralise the endosomal pH. An alternative explanation to the proton sponge effect is that complexes are released from lysosomes through small membrane damage, and that the lysosome in fact stays intact [112]. Overall, whatever the actual mechanism of this ‘sponge effect’ polymers with high buffering capacity, such as PEI have a beneficial effect on endosomal escape.

Other common polycations such as PLL lack this ability to facilitate endosomal escape [113]. One way of addressing this issue is through the introduction of lysosomotropic reagents such as chloroquine to DNA polyplexes [114]. This method has successfully increased the transfection efficiency of PLL based delivery systems [115], [116]. However, it has been found that these lysosomotropic reagents are also toxic [117], possibly due to the increased membrane permeabilisation that they cause [118]. As such, this method of delivery is limited in its application.

An alternative strategy to improve endosomal escape is through the introduction of histidine residues to the polymer [119]. It is thought that histidine can successfully increase transfection efficiency acting again as a proton sponge within the polyplex. This is due to the imidazole present in histidine that has a pK_a_ of ∼6 meaning that in slightly acidic media of the endosome it is will become protonated. Evidence for the success of this process can be seen through its escape from negatively charged liposomes in acidic media [120].

Niidome et al. employed this strategy to prepare novel PLL dendrimers. While no beneficial effect was observed for histidine capped-PLL polyplexes prepared at neutral pH, a significant increase in their transfection efficiency was observed when the histidine containing polyplexes where prepared at pH 5. The authors demonstrated that the latter polyplexes were more stable upon incubation in buffer, demonstrating the role of histidine protonation in polyplex formation.

## 4 Other biomedical applications of PIC particles

While gene delivery has been possibly the field where PIC particles have made the biggest impact, there are other areas where these particles have had a significant contribution [17]. In this section we will describe representative examples of the application of PIC particles for the encapsulation and delivery of proteins and small molecules. Interested readers are referred to specialised reviews [5], [6], [7], [8].

Proteins and polysaccharides are other biomacromolecules that often present multiple charges in their backbone. Chitosan, alginate or hyaluronic acid are charged polysaccharides that have been commonly employed as biocompatible polyelectrolytes in the formulation of PIC particles [121]. They often play a passive/structural role, although their enzymatic degradation has been exploited to trigger the release of encapsulated materials [122]. Proteins however tend to have an active role and researchers have been interested in the application of PIC particles to protect and deliver ‘functional’ proteins such as enzymes [51]. When compared to polysaccharides and nucleic acids, proteins normally present a lower density of charges. Moreover, the presence of positively and negatively charged residues complicates things further and the overall charge of the protein will depend on their isoelectric point and the pH at which the formulation has to be prepared.

A good example of the challenges in the preparation of PIC particles from functional proteins has been described by Giannotti et al.*,* who prepared PIC particles from *N*-trimethyl chitosan and the anionic lysosomal enzyme α-galactosidase A (α-GalA) [51]. These complexes only formed at pH values between 7.3 and 8.0, whereas no particles formed at pH 4.5–5.5, as expected from the neutralisation of α-GalA at pH values close to its isoelectric point (pI ∼ 5.7). Similarly, these nanoparticles disassembled at acidic pH, which could be exploited to trigger enzyme release inside the lysosomes, where the authors found these complexes are accumulated *in vitro*. Although a fair 62% of the initial activity of α-GalA was retained after complexation into PIC particles, there is still room for improving this result and develop complexes that do not affect the complexed enzyme and maintain its full activity.

More challenging is the encapsulation of small peptides, which very often have few charge residues and thus weak charge densities, making their complexation into PIC particles particularly unfavourable (see Section 2.1). This is the case for insulin, whose molecular weight is almost 15 times smaller than that of the enzyme α-GalA discussed above. Despite this intrinsic limitation, Jintapattanakit et al. complexed acidic insulin with *N*-trimethyl chitosan into PIC particles, which they evaluated as potential vehicles for the oral delivery of insulin [123]. The oral administration of peptides is limited by their degradation by endogenous proteases. However, the authors found that the stability of free insulin to digestion by trypsin, used as a model gastric protease, was almost doubled when the peptide was complexed inside PIC particles. However, a recent report highlights how despite increasing the stability to proteases, it is the release and subsequent absorption of insulin in the gastrointestinal tract that currently limits the efficacy of PIC particles for this application [124]. Alternative ways of stabilising and administering such complexes must be explored to circumvent this limitation.

The complexation of short charged peptides with very few charges can be facilitated by the addition of cross-linkers, which allow peptide polymerisation and thus increase their binding affinity (Section 2.7). Our group has recently exploited this strategy for the targeted delivery of antimicrobial PEI (Fig. 10) [68]. Incorporation of cysteine residues into a short peptide sequence allowed self-assembly with as little as three negative charges per peptide, otherwise not possible. The peptides were designed to incorporate a short sequence that was cleavable by *Pseudomonas aeruginosa*’s elastase. When these nanoparticles were exposed to the bacterial elastase, the cross-linked peptides were hydrolysed, reducing this way charge density, thus causing particles to disassemble and release the PEI. PIC particles were stable in the absence of this bacterial elastase, and were not able to elicit an antimicrobial effect on a *P. aeruginosa* mutant that was unable to produce this enzyme.

More challenging is the complexation of singly charged molecules such as the chemotherapeutic doxorubicin (DOX) that presents a single amine group in its structure. Hsieh et al. prepared DOX-loaded PIC particle system from PAA/PLL blends, cross-linking the amines in PLL with genipin [56]. In this paper, the authors illustrate how the release kinetics of DOX (or virtually any loaded drug) could be tuned by changing the degree of cross-linking, thus changing the permeability of the nanoparticles. In addition, the weak polyelectrolyte PAA endowed PIC particles with pH-responsive behaviour, accelerating the release of DOX under acidic conditions.

Alternatively, PIC particles can have a “passive” role in the delivery of small molecules, where electrostatic interactions between the polyelectrolytes and the drug to be delivered do not play a role in the encapsulation. For example, non-ionic chemotherapeutic paclitaxel has been encapsulated into the hydrophobic core of PIC particles prepared in this case from hyaluronic acid and styrylpyridinium [125]. The UV cross-linking of styrylpyridinium in the core stabilised the assembled particle, which was then loaded with paclitaxel. High cellular uptake and sustained drug release were achieved from these nanoparticles, proving the utility of PIC particles as vehicles for non-charged drugs as well.

## 5 Conclusions and outlook

This review gives an overview of the preparation and biomedical applications of PIC particles. A comprehensive description of the factors affecting PIC particle self-assembly has been included: Here, we highlight the effect of polyelectrolyte molecular weight, concentration and charge density, pH and ionic strength of the (aqueous) media, or relative ratio and mixing order of both polyelectrolytes, in particle preparation and stability. Understanding how to optimise PIC particle preparation and stability is of key importance for the development of biomedical application of these nanomaterials. Representative examples of their application in gene, protein and small molecule delivery have been included to illustrate the particular challenges faced in these applications, in particular the issues surrounding toxicity and transfection efficiency in gene delivery. Selected examples of the use of targeted and responsive delivery systems have been included, which highlight how polymer synthesis and characterisation can allow the field of PIC particles, as well as other polyelectrolyte complex nanomaterials, to eventually develop truly mimics of natural delivery systems.
